# Spontaneous renal hemorrhage in acquired cystic kidney disease

**DOI:** 10.1002/jgf2.400

**Published:** 2020-11-24

**Authors:** Masato Kajikawa, Hidehiro Tanji, Yukihito Higashi

**Affiliations:** ^1^ Division of Regeneration and Medicine Medical Center for Translational and Clinical Research Hiroshima University Hospital Hiroshima Japan; ^2^ Department of Surgery Yoshida General Hospital Akitakata Japan; ^3^ Department of Cardiovascular Regeneration and Medicine Research Institute for Radiation Biology and Medicine Hiroshima University Hiroshima Japan

**Keywords:** acquired cystic kidney disease, spontaneous renal hemorrhage

## Abstract

We performed computed tomography every year and pointed out the development of kidney atrophy and a cystic lesion in relation to prolonged hemodialysis, which may be cause of spontaneous renal hemorrhage.

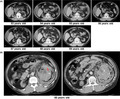

A 60‐year‐old man was admitted to the hospital for sudden onset of severe left abdominal pain without any episode of trauma. He had end‐stage renal disease caused by hypertensive nephrosclerosis and had been undergoing peritoneal dialysis treatment for 7 years. Development of kidney atrophy and a cystic lesion in relation to prolonged hemodialysis had been pointed out (Figure 1A). He was converted to maintenance hemodialysis with heparin anticoagulation 1 year earlier. His home blood pressure was not controlled well despite taking angiotensin II receptor blocker, calcium channel blocker, and beta‐blocker. On admission, his vital signs were as follows: blood pressure, 106/76 mm Hg; heart rate, 78 bpm; body temperature, 34.8°C. Laboratory investigations showed the following: hemoglobin, 9.5 g/dL; C‐reactive protein, 1.13 mg/dL. Abdominal computed tomography at the hospital emergency room showed intracystic hemorrhage (Figure 1B, red arrow) and marked distention of the left kidney (Figure 1B, white arrowheads) with massive parenchymal and perirenal hemorrhage (Figure 1B, white arrows). He had not been taking antiplatelet or anticoagulant agents. He was diagnosed with spontaneous renal hemorrhage in the left kidney. Nephrectomy of left kidney and red blood cell transfusion was performed. Pathological findings revealed nonmalignant parenchymal hemorrhage of the kidney. After nephrectomy, he resumed daily life. Chronic renal failure is associated with the development of acquired cystic kidney disease.^1^, ^2^ The prevalence of acquired cystic kidney disease increases with longer duration of maintenance hemodialysis or peritoneal dialysis.^1^ Acquired cystic kidney disease is characterized by the development of multiple and bilateral renal cysts, which are usually <0.5 cm in diameter and are rarely greater than 2 to 3 cm. Most patients with acquired cystic kidney disease are asymptomatic. However, some patients may present with hematuria, lumbar pain, urinary tract infection, and development of malignancy.^2^ Although it is rare and unknown etiology, spontaneous renal hemorrhage in acquired cystic kidney disease is a cause of acute abdomen.

**Figure 1:**
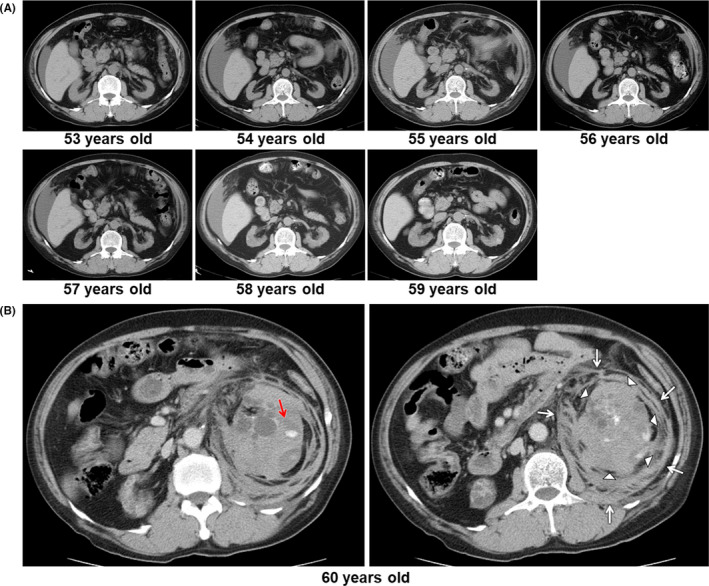
Development of kidney atrophy and a cystic lesion (A); intracystic hemorrhage (B, red arrow); distention of the left kidney (B, white arrowheads); perirenal hemorrhage (B, white arrows)

## CONFLICT OF INTEREST

The other authors have stated explicitly that there are no conflicts of interest in connection with this article.

## INFORMED CONSENT

Written informed consent for publishing this case report was obtained from patient.
